# A ground truth data set of gas chromatography mass spectrometry (GCMS) analysed synthesised methylenedioxymethylamphetamine (MDMA)

**DOI:** 10.1016/j.dib.2023.108931

**Published:** 2023-01-25

**Authors:** Jonathan Miller, Roberto Puch-Solis, Hilary Ann Scott Buchanan, Niamh Nic Daeid

**Affiliations:** Leverhulme Research Centre for Forensic Science, School of Science and Engineering, University of Dundee, Nethergate, Dundee DD1 4HN, Scotland, United Kingdom

**Keywords:** MDMA, GCMS, Statistical modelling data, Machine learning data

## Abstract

Controlled drug samples are normally chemically analysed to determine their identity and in some cases, their purity. There are also circumstances where a more broad chemical characterisation of drug samples may also be required. This involves investigating the chemical impurities that may be present in a drug sample as a consequence of their synthesis. This impurity or drug profiling can be derived from drugs which are synthesised chemically or extracted from plant materials and then modified chemically. Impurity profiling can provide some insight into the synthetic methods used and sometimes the starting chemicals used. We report on the data generated from repetitive (n=18) synthesis of ecstasy (methylenedioxymethylamphetamine or MDMA) made by three different synthetic methods. Each data sample is expressed in multiple formats.

This article uses the template for publishing GCMS data provided in Miller et al.(2022)[1]. The template provides a robust and systematic approach to organise GCMS data that is both useful for practitioners and amenable for automated data manipulation by data scientists.

**Specification Table**

**Table:** 

Subject	Analytical Chemistry
Specific subject area	Collection of chromatographic impurity profiles for a series of synthesised samples of methylenedioxymethylamphetamine (MDMA) prepared using three reductive amination synthetic pathways.
Type of data	Binary (Raw)
	Table
	Graph
	Report
How data were acquired	Data acquisition occurred in three distinct phases. Phase I involved the repetitive synthesis (n=6) of MDMA using three reductive amination synthetic methods resulting in 18 samples. In phase II the data was processed and analysed using an Agilent gas chromatography mass spectrometry (GCMS) Hewlett-Packard (HP) 6890/5973 MS Chemstation (version B.00.01 Hewlet Packard, Agilent Technologies), following ASTM E1618 protocols. In Phase III the data was engineered using Agilent’s proprietary software and open source software: OpenChrom 1.4 [2], Julia 1.7 [3] and Linux (Ubuntu 21.04) [4].
Data format	Raw, Analysed, Filtered
Parameters for data collection	GCMS standard operating procedures were adhered to for sample analysis, including temperature, run times, carrier gas and internal standards. Samples were grouped into classifications based on the reductive amination synthetic method used in their production and sample batch number (1–6).
Description of data collection	Samples were prepared in house following literature methods. Analysis was undertaken using GCMS to provide chromatographic data in relation to the chemical impurities associated with each method of preparation. Mass spectrometry and chromatography information was extracted via standard GCMS techniques. Outputted data was programmatically engineered into accessible formats using open source software. Isotope ratio mass spectrometry data is also available for these samples [2]
Data source location	Institution: University of Dundee City: Dundee Country: Scotland, UK
Data accessibility	Repository name: Discovery research portal - https://discovery.dundee.ac.uk/
	Data identification number: https://doi.org/10.15132/10000184
	Direct URL to data: https://doi.org/10.15132/10000184
	Instructions for accessing these data: The dataset (870 MB) is stored online as a ZIP file.
Related research article	Buchanan, H. A. S.; NicDaeid, N.; Meier-Augenstein, W.; Kemp, H. F.; Kerr, W. J.; Middleditch, M., Emerging use of isotope ratio mass spectrometry as a tool for discrimination of 3,4-methylenedioxymethamphetamine by synthetic route. Analytical Chemistry 2008, 80(9), 3350–3356 [5].

## Value of the Data

• Drug impurity profiling data provide opportunities for the assessment of potential sample to sample linkages of synthesised and semi-synthesised controlled substances. This can reveal intelligence information about the synthetic methods used to prepare the substances. The data presented can be considered as a ground truth data set where the provenance of the data is known and each sample is the result of carefully controlled synthetic methods by a single chemist.
• The data is beneficial to forensic chemists who are tasked with developing methods associated with impurity profiling of MDMA/Ecstasy samples. The data sets also enable the development of algorithmic processes for pattern matching between impurity profiles derived from seized MDMA samples. Ultimately the provides opportunities for the development of intelligence information in clandestine production of MDMA.
• The data provides a basis upon which further chemical impurity profiles from MDMA can be added which could explore reproducibility of the synthetic and analytic methods between chemists and analysts. Such additions will develop further the knowledge base for the importance and utilisation of chemical impurity profiling of MDMA and the development of machine learning methods in this area.

## 1 Objective

This work provides a foundational dataset of analytical information which identifies chemical products that can be used to characterise synthetic pathways used for the preparation of Ecstasy. The data is of known provenance as the compounds from which it was generated were synthesised by the authors. As such the data was designed to add value to the knowledge base in this area. The data complements previous interpretations [5] undertaken by some of the authors.

## 2 Data Description

MDMA was synthesised from phenylmethylketone (PMK) following three synthetic reductive amination methods:

1. Mercury amalgam (Al/Hg)
2. Sodium borohydride (NaBH${}_{4}$)
3. Platinium hydride (Pt/H${}_{2}$)

**Fig. 1:**
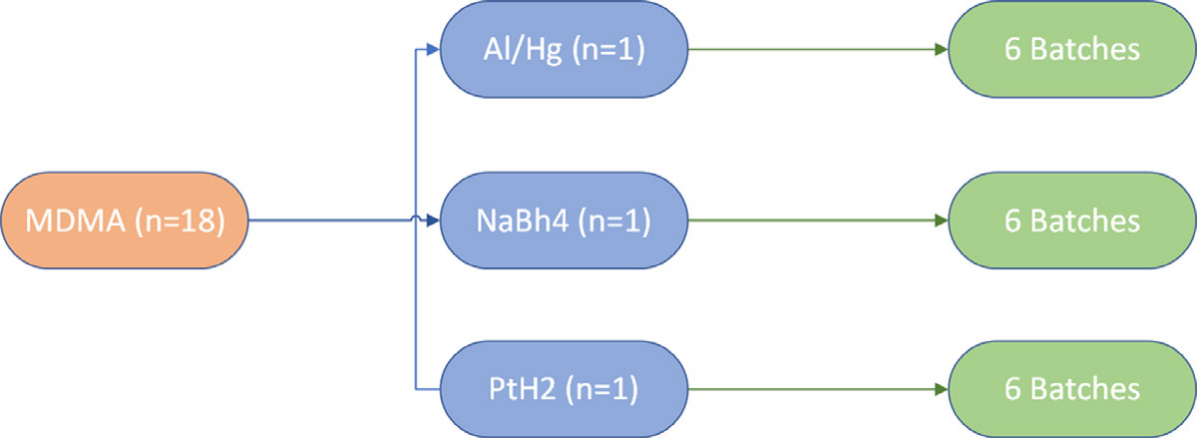
Pathways for MDMA synthesis and batch production.

In each case six repetitive batches were prepared by the same chemist using the same materials and glassware, resulting in 18 samples in total, Fig. 1.

**Fig. 2:**
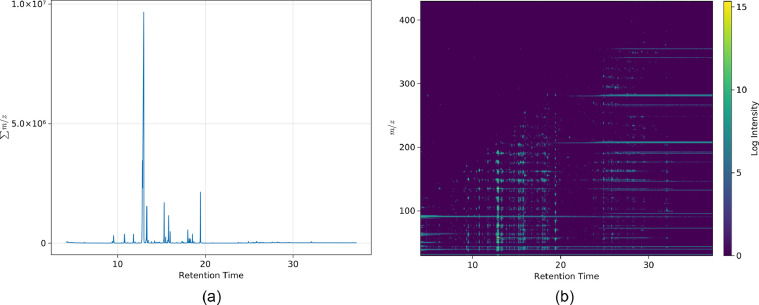
Visualisation of MDMA made with the Al/Hg based method. (a) The plot for the total ion chromatogram, (b) the heat map for the total ion spectrum.

A chromatogram and a heat map were produced for each sample, Fig. 2. As the samples contain an internal standard (ISTD) - a known amount of a chemical added to each sample to facilitate the determination of the concentration of the chemicals contained within the sample - which produces an ISTD peak, one additional plot was produced for each sample, with (a) the ISTD peak present and (b) the ISTD peak removed.

The data set was organised into folders where each folder contained a single sample. Each sample folder was assigned a unique name which contained the defining information of the sample. This prevents data from being allocated to the wrong place while enabling management of the data programmatically. An example of a sample folder name is:

DrugSamples_MDMA_AlHg_Batch-75C.D where,

1. “DrugSamples”. Prefix that is the same for all sample folders that identifies the data set as drug samples.
2. “MDMA”. This is the classification of the drug samples. There is only one value.
3. “AlHg”. This is the route taken to synthesise the MDMA. Possible values are: “AlHg”, “NaBH4” and “PtH2”.
4. “Batch-75C”. This is the batch number. Possible values depends on the route taken. For “AlHg” they are “Batch-75C”, “Batch-76C”, “Batch-78C”,…, “Batch-81C”; for “NaBH4”, “Batch-101”,…, “Batch-106”; and for “PtH2”, “Batch-112”, “Batch-113”, “Batch-116”, “Batch-117”, “Batch-119”, “Batch-121”.

Each sample folder contains raw data, figures, tables and reports. The folder name storing all of the files associated with the same sample should be the same as the sample name. The name of each file within the folder, to protect data integrity, should also contain the sample name with the exception of any raw data files produced by instrument software. These files are required to be readable by OpenChrom in order that the analytical output can be accessed independently of the GCMS instrument. In the data presented, the GCMS instrument software was Agilent Chemstation software which produces the files “ANALYTICALMETHODS_MDMA.M”, “DATA.MS”, “GC01A.CH” and “PRE_POST.INI” for each sample analysed. OpenChrom was used to produce a range of files for each sample which were saved into the sample folder. The method for producing these files using OpenChrom are described in the experimental design.

**Fig. 3:**
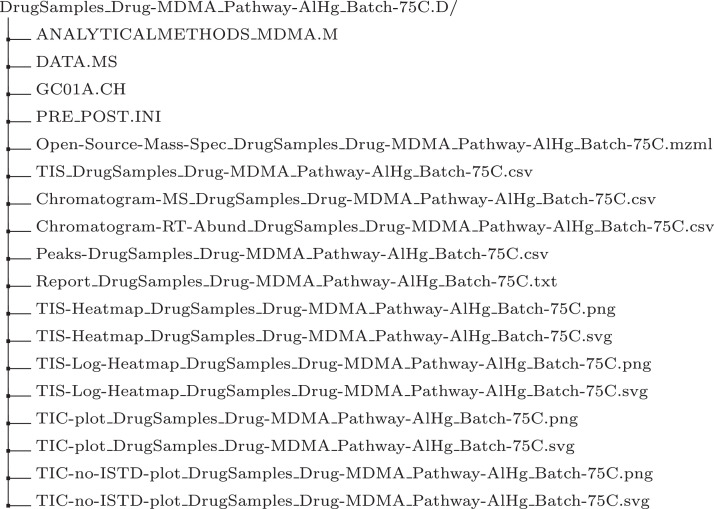
An example of a sample folder structure.

Using these conventions, the tree directory for the MDMA sample is given in Fig. 3. The description of each file according to its prefix is given below.

1. “Open-Source-Mass-Spec_”. Agilent, by default, produces GCMS data in its proprietary format with data folders ending in “.D”. This file is the open-source equivalent [6].
2. “TIS_”. This file contains all the information required to visualise the chromatogram and heat map. It is a table with column names “RT(milliseconds)”, “RT(minutes) - NOT USED BY IMPORT”, “RI” relative intensity and the column is an empty delimiter between RTs and m/z to intensities. It then has a variable number of columns with positive integer value names usually starting from “30” and progressed by increments of one, e.g. “31”, “32”,..., “429”. Columns “RT(milliseconds)” and “RT(minutes) - NOT USED BY IMPORT” contain retention times in milliseconds and minutes. The retention times are recorded in intervals on an average of 600 milliseconds that is exported from OpenChrom.
3. “Chromatogram-MS_”. This file contains data processed from “TIS_” to ease the production of the heat map. Only the TIS matrix with row and column headers is present. The retention time column is labelled “RetentionTimeMin”, and the m/z columns are labelled as in “TIS_”.
4. “Chromatogram-RT-Abund_”. This file contains data processed from ”TIS_” to ease the production of a chromatogram. Only the TIC value pairs are present. There are two columns, the retention time and the sum of the m/z intensities, titled RetentionTimeMin and Abundance respectively.
5. “Peaks_”. This file consists of a table containing detected peaks, which are used to aid the determination of chemical composition. The table contains columns “RT [min]”, “Area” and “m/z” used to aid in the identification of a sample. The other columns present are automatically produced by OpenChrom and are not relevant for sample identification.
6. “Report_”. This is a general report of the sample peaks that contains meta data for the sample. It includes operator’s name (Operator) and the sample and (Data Name).
7. “TIS-Heatmap_”. This prefix refers to two figures, in PNG and SVG formats, displaying a heat map produced from values in “Chromatogram-MS_” with no transformation.
8. “TIS-Log-Heatmap_”. This prefix refers to two figures, in PNG and SVG formats, displaying a heat map produced from values in “Chromatogram-MS_” with a natural logarithmic (base e) scale transformation.
9. “TIC-plot_”. This prefix refers to the TIC plot created from the file with prefix “Chromatogram-RT-Abund_”. The figure is produced in SVG and PNG formats. The figures are ready for inspection and can be used for automated analysis.
10. “TIC-no-ISTD-plot_”. This prefix refers to the TIC plot where the ISTD peak has been removed. The figure comes in PNG and SVG formats. ISTD peak is not informative for identification.

## 3 Experimental Design, Materials and Methods

The data was produced in three phases. Phase I involved synthesis of the 18 MDMA samples, phase II involved the analysis of the synthesised MDMA to generate a chemical impurity profile associated with each sample, and phase III consisted of processing the data produced in phase II to transform it into a set of formats usable for further data analysis.

### 3.1 Phase I: chemical synthesis

Chemical synthesis was undertaken in a number of steps. Firstly, a common precursor material, phenylmethylketone (PMK) was prepared from safrole via isosafrole and isosafrole glycol as intermediate compounds. PMK was then used as the starting material for the preparation of the target MDMA using three different synthetic pathways (i) Al/Hg amalgam reductive amination; (ii) NaBH${}_{4}$ reductive amination; (iii) Pt/H${}_{2}$ reductive amination. Each synthetic method produced MDMA as a freebase (which is an oil) and was converted to the corresponding hydrochloride salt (a white solid) in each case. A full synthesis is provided in Buchanan et al. [5].

Six repeat batches of MDMA (salt form) from each of the three synthetic reductive amination reaction pathways using the same chemicals and glass wear and by the same chemist were prepared to give 18 batches in total: six Al/Hg amalgam (ALHg-75, 76, 78,...,81), six NaBH${}_{4}$ (NaBH-4101,...,105), and six Pt/H${}_{2}$ (PtH2-112, 113, 116, 117, 119, 121).

### 3.2 Phase II: chemical analysis

Chemical analysis of the samples produced in phase I involved two steps. Firstly, the chemical impurities associated with the synthetic method were solvent extracted from MDMA. This process involved dissolving MDMA in a solvent and adjusting the pH of the resultant solution followed by a second solvent extraction to preferentially extract the impurities [6]. An internal standard of known concentration was also added to the extracted impurity solution in preparation for analysis. Secondly, the samples containing the extracted impurity profiles were analysed using gas chromatography mass spectrometry (GCMS) in accordance with the published method [6]. Reproducibility of the extraction and repeatability of the GCMS analysis were assessed (Tables 1 and 2).

**Table 1:** Reproducibility of the extraction.

Peak	RSD area

1	12.9%
2	10.0%
3	14.0%
4	13.9%
5	9.3%
6	5.1%
7	13.8%
8	18.9%
9	1.8%
10	14.8%
Avg	11.5%

**Table 2:** Repeatability of the GCMS analysis of one extracted sample.

Peak	RSD area
1	2.0%
2	3.2%
3	1.0%
4	1.2%
5	2.3%
6	1.5%
7	8.8%
8	4.1%
9	2.0%
10	1.8%
Avg	2.8%

#### 3.2.1 Reproducibility of the extraction

The within day reproducibility of the sample extraction was assessed across the peak area response of 10 impurity peaks normalised to (divided by) the peak area of the internal standard within the same sample extract. Normalised peak areas were compared across six different extractions of the same batch of MDMA (Table 1). Extractions and analyses were undertaken on the same day. The relative standard deviation of the normalised peak areas show good reproducibility across the sample extracts (average RSD = 11.5%) in line with other published values [6].

#### 3.2.2 Repeatability of the GCMS analysis of one extracted sample.

The peak area response of 10 impurity peaks in one extracted sample were normalised to the peak area of the internal standard across six repetitive GCMS analysis of the same sample extract (Table 2). Extractions and analyses were undertaken on the same day. The relative standard deviation (RSD) of the normalised peak areas show very good repeatability (average RSD =2.8%).

### 3.3 Phase III: data engineering

In phase III, OpenChrom [7] was used to process the Agilent proprietary data into open source formats. OpenChrom is an open source program developed to view and analyse chromatographic as well as other types of data. Specifically OpenChrom can open GCMS data acquired from most proprietary vendors and runs on macOS, Windows and Linux [2].

In addition, in Phase III, a software suite, Automated Mass spectral Deconvolution and Identification System (AMDIS), was used to identify compounds in GCMS data [8]. The program deconvolutes the data to separate individual compounds into local peaks. AMDIS is only available on Windows. To run AMDIS on Linux, users are recommended to use a Windows emulator, such as *wine* [9]. Users on Apple computers are recommended to use a virtual machine, such as VirtualBox [10], where Windows can be installed.

The processes in this section were completed in Linux. The following steps were applied to each sample. The steps are the same as in Miller et al. [1] but adapted for the dataset presented in this article.

1. The GCMS ChemStation software names the sample folders it produces with numbers, e.g. “12345678.D”. The sample folders were rename according to the naming conventions introduced above, Fig. 3, e.g. DrugSamples_Drug-MDMA_Pathway-AlHg_Batch-75C.D.
2. OpenChrom can read the sample data folder generated by ChemStation without any modification.
3. OpenChrom detect peaks using the sample data and the AMDIS database, which must be installed. OpenChrom detects peaks as follows:
  (i) right-click on the chromatogram,
  (ii) move the mouse pointer over “Peak Detector” and
  (iii) click “AMDIS (extern)” from the menu.
  This produces a number of windows. The peaks are stored in memory and an inverted triangle is displayed on top of the detected peaks.
4. Peak areas were obtained in OpenChrom following the steps:
  (i) right-click on the chromatogram,
  (ii) hover over “Peak Integrator”,
  (iii) select “Peak Integrator Trapezoid”; a window with title “Edit Processor Options” appears,
  (iv) keep the default button highlighted “Use System Options”, and
  (v) select “Finish” at the bottom right corner of the window.
5. The calculated retention times and areas were recorded in a comma separated value (CSV) file following the naming convention in Fig. 1, and using prefix “Peaks_”. To record the detected peaks and areas,
  (i) right-click on the chromatogram,
  (ii) move the cursor over “Peak Export”,
  (iii) select “CSV Peak Export (*.csv)”, a window with title “Edit Processor Options” will pop up with radio button “Use Specific Options” already selected; leave it selected,
  (iv) choose the location of the folder to record the CSV file by selecting the button inline with “Export Folder” and to the right-side of the window, the button is rectangular with a single ellipsis,
  (v) write the CSV file name in the box labelled “Filename” following the naming convention, i.e. with prefix “Peaks_”,
  (vi) click “Finish” at the bottom right of the pop-up window.
6. OpenChrom created a report that contains the sample information and the peak areas. The report was saved in the file with prefix “Report_” in text format (with extension “*.txt”); the report is obtained following the steps,
  (i) right-click on the chromatogram,
  (ii) select “Chromatogram Reports”,
  (iii) select “OpenChrom Report (*.txt)”,
  (iv) save the report by following the same process as in item 5, and by replacing prefix “Peaks_” with “Report_”.
7. To record the TIS as a CSV file,
  (i) right-click on the chromatogram,
  (ii) move the cursor over “Chromatogram Export”,
  (iii) click on “CSV Chromatogram (*.csv)”
  (iv) and follow the same process as in item 5, replacing the prefix with “Chromatogram-MS_”.
8. At this point the sample file names are of the form DrugSamples_Drug-MDMA_Pathway-AlHg_Batch-75C.D, where the last block after the “_” represents the batch number of the drug production. For example, the file name in item 1 in this list contains “Batch-75C” meaning that it is batch 75C.
9. The sample file with prefix “Chromatogram-MS_” used to calculate a row wise summation of the m/z ion columns. The calculation produced the abundance at each time step as recorded by ChemStation. A CSV file consisting of the retention time and abundance was saved to a file with its name prefixed with “Chromatogram-RT-Abund_”, which contains the data for creating the TIC.
10. Each sample contained an internal standard which generated an ISTD peak. An extra chromatogram was created with the ISTD peak removed. It was removed by setting its retention time to 0. The file names encoded this information with the prefixes “TIC-plot_” and “TIC-no_ISTD-plot_”. Each plot was recorded in SVG and PNG formats.
11. The file produced from item 7 in this list was modified by removing extraneous columns and a new CSV file was recorded with prefix “Chromatogram-MS_”.
12. The raw and log-valued TIS data was visualised using the prefixed file “Chromatogram-MS_” and saved to file with prefixes “TIS-Heatmap_” and “TIS-Log-Heatmap_”. Both were saved in SVG and PNG formats.

## Ethics Statement

There were no ethical requirements for the collection and analysis of the data. All software used for the curation and analysis of the dataset were open source.

## CRediT authorship contribution statement

**Jonathan Miller:** Data curation, Software, Writing – original draft. **Roberto Puch-Solis:** Supervision, Writing – original draft. **Hilary Ann Scott Buchanan:** Conceptualization, Data curation, Methodology. **Niamh Nic Daeid:** Conceptualization, Methodology, Funding acquisition, Supervision, Writing – review & editing.

## Declaration of Competing Interest

The authors declare that they have no known competing financial interests or personal relationships which have, or could be perceived to have, influenced the work reported in this article.

## Data Availability

A Ground Truth Data Set of GCMS Analysed Synthesised MDMA (Original data) (discovery@dundee.ac.uk).

## References

[1] J. Miller, R. Puch-Solis, W. N. S. Mat-Desa, N. Nic Daeid, A UK-based ground truth data set of GCMS analysed ignitable liquid samples—A template for making chromatographic data accessible as an open source data set (submitted). (2022). 10.1016/j.dib.2022.108670 PMC9679696 36425998

[2] Wenig P., Odermatt J. (2010). Openchrom: a cross-platform open source software for the mass spectrometric analysis of chromatographic data. BMC Bioinform..

[3] Bezanson J., Edelman A., Karpinski S., Shah V.B. (2017). Julia: a fresh approach to numerical computing. SIAM Rev..

[4] Ubuntu, Download ubuntu desktop, 2004, Visited: 2022-03-31 [Online], https://ubuntu.com/.

[5] Buchanan H.A.S., Daid N.N., Meier-Augenstein W., Kemp H.F., Kerr W.J., Middleditch M. (2008). Emerging use of isotope ratio mass spectrometry as a tool for discrimination of 3,4-methylenedioxymethamphetamine by synthetic route. Anal. Chem..

[6] Deutsch E.W. (2010).

[7] Lablicate, Openchrom, 2010, Visited: 2022-01-01 [Online], https://www.lablicate.com/.

[8] AMDIS, CHEMDATA.NIST.GOV Mass Spectrometry Data Center, 2004, Visited: 2022-01-30 [Online], https://chemdata.nist.gov/dokuwiki/doku.php?id=chemdata%3Aamdis.

[9] Wine, Wine what is wine?, 1993, Visited: 2022-01-30 [Online], https://www.winehq.org/.

[10] Virtual Box, Welcome to VirtualBox.org!, 2010, Visited: 2022-03-31 [Online], https://www.virtualbox.org/.

